# Glossography – a computer vision technique for assessing involuntary tongue movements in dyskinesias

**DOI:** 10.3389/fneur.2026.1758570

**Published:** 2026-03-20

**Authors:** Maksymilian Aleksander Brzezicki, Przemyslaw Temistokles Zakowicz, Marcin Palczynski, Emilia Pilchowska-Ujma, Olga Szymańska-Adamcewicz, Krystian Kamil Pietrykowski, Natalia Pawlak, Pawel Lesniak, Szymon Jurga

**Affiliations:** 1 Jesus College, University of Oxford, Oxford, United Kingdom; 2 Department of Neural Engineering and Space Medicine, Institute of Medical Sciences, University of Zielona Góra, Zielona Gora, Poland; 3 Centre for Space Research, Polish Academy of Sciences, Warsaw, Poland; 4 Department of Neurology, University of Zielona Gora, Zielona Gora, Poland

**Keywords:** computer vision, dyskinesias, machine learning, neurodegeneration, Parkinson’s disease

## Abstract

We present a novel computer vision approach using DeepLabCut to objectively quantify orofacial dyskinesias in Parkinson’s disease. Unlike traditional wearable sensors that focus on limb movements, this method tracks tongue, chin, nose, and forehead landmarks through standard video recordings using a fully markerless deep learning pipeline, capturing metrics including displacement, variability, and peak movements. Analysis of a hospitalised patient over 4 days demonstrated progressive reduction in dyskinetic parameters correlating with medication adjustments, consistent with concurrent clinical assessment using the Unified Dyskinesia Rating Scale (UDysRS) and modified Abnormal Involuntary Movement Scale (mAIMS). Though resource-intensive, glossography offers potential for remote monitoring in underserved areas with limited specialist access. The technique provides granular movement assessment using widely available technology, potentially enhancing treatment precision beyond traditional clinician-administered rating scales.

## Introduction

Current assessment of dyskinesias in Parkinson’s disease (PD) relies heavily on neurological examination with clinician-administered rating scales like the Unified Dyskinesia Rating Scale (UDysRS) (1) and modified Abnormal Involuntary Movement Scale (mAIMS) (2). While these scales have clinical utility, they may lack capacity for continuous, granular monitoring of movement patterns (3). This is of particular interest for patients with advanced PD who may require personalised medication adjustments with a narrowing levodopa therapeutic window. Wearable sensor systems have emerged as objective measurement tools, using accelerometry-based algorithms to quantify dyskinesia severity through continuous monitoring (4). However, current sensor technologies focus predominantly on limb movements and require specialised limb-worn equipment, with no capacity to capture critical orofacial manifestations of dyskinesia involving facial and tongue muscle movements. Computer vision systems may bridge this gap, offering higher granularity of assessment with even a simple phone camera that can record movements at the bedside.

The assessment of tongue movement disorders currently relies almost exclusively on clinical observation and subjective rating scales. Electromyography (EMG) can record muscle activity but does not capture spatial kinematics. Ultrasound has been used in research settings to image tongue musculature but is operator-dependent and not suited to continuous monitoring. Electromagnetic articulography (EMA) represents the current reference standard for tongue movement measurement in research, providing millimetre-resolution tracking, but is expensive, requires highly specialised infrastructure, and cannot be used in routine clinical practice. Glossography, as presented here, addresses this gap using widely available video technology and a fully automated, markerless tracking approach.

## Methods

### Patient and setting

The patient was admitted to the Department of Neurology at the Green Mountain University Hospital in Zielona Gora, a tertiary referral centre for neurological diseases in western Poland. Movements were recorded daily for the duration of the hospital stay (4 days) to capture changes in response to medication schedule adjustments. Orofacial symptoms were the patient’s predominant concern, limiting quality of life and activities of daily living. Concurrent clinical assessment was performed by the attending neurologist using the UDysRS and mAIMS at each daily evaluation. The patient provided prior written informed consent for publication of all data and images, including facial images, in accordance with EU research integrity regulations.

### Video recording and landmark selection

**Figure 1:**
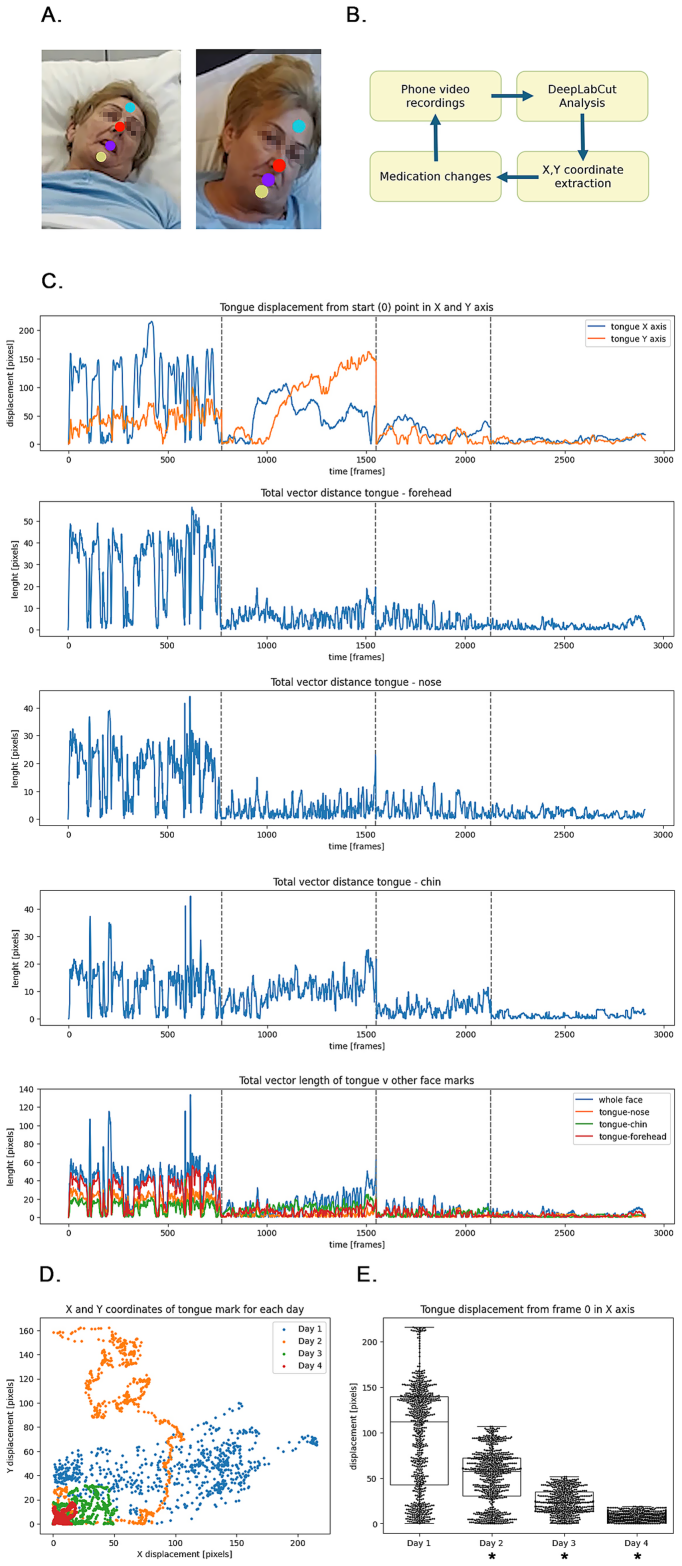
Summary of glossographic analysis. **(A)** Orofacial landmarks defined for training of the computer vision model using the standard DeepLabCut protocol. Colors indicate individual markers for forehead, nose, tongue tip, and chin. Patient facial image has been blurred for anonymization. **(B)** Clinical pipeline for the use of the algorithm in daily decision-making for medication adjustments. **(C)** Tongue displacement from index frame in X and Y axes (upper panel); vector distances between the tongue and respective orofacial landmarks (lower panels). Grey dashed vertical lines indicate the start of each new day. **(D)** X and Y coordinates for tongue displacement during respective admission days. **(E)** Box-and-whisker plots for tongue displacement in the X axis; a dash indicates *p* < 0.001; one-way ANOVA with *post-hoc* Tukey test.

Thirty-second video segments were recorded at the bedside on each day of admission using a standard camera. Four orofacial landmarks were defined for tracking: tongue tip, chin, nose, and forehead (Figure 1A).

### DeepLabCut markerless pose estimation

Landmark tracking was performed using DeepLabCut (5), an open-source framework for markerless pose estimation based on transfer learning with deep convolutional neural networks. No physical marker or sensor is placed on the patient. The workflow comprises two stages. First, a researcher manually annotates the four orofacial landmarks on a small number of representative frames (approximately 200 frames drawn from the video dataset) using the DeepLabCut graphical interface. DeepLabCut leverages a pre-trained ResNet deep neural network (transfer learning) and fine-tunes it on these labelled frames, learning the visual features associated with each landmark. Once trained, the network automatically predicts the pixel coordinates (X and Y) of each landmark in every frame of the remaining videos, achieving accuracy comparable to human annotators. All videos were reviewed following analysis to confirm correct landmark placement by the trained network. The clinical pipeline is illustrated in Figure 1B.

### Feature extraction and statistical analysis

X and Y coordinates were extracted for the tongue tip marker (Greek: glossa γλώσσα, hence: glossography), and displacement from the first (index) frame was calculated (Figure 1C). Vector distances between the tongue and each of the remaining orofacial landmarks were calculated using the Euclidean norm, with whole-face total vector length defined as the sum of all such vectors (Figure 1C, lowest panel). Discrete dyskinetic movement peaks were identified using the SciPy Signal Find Peaks algorithm. Movement variability was assessed using standard deviation and Poincaré analysis (SD1 and SD2). Data were tested for normality by visual inspection, with group differences assessed using one-way ANOVA with *post-hoc* Tukey test (Figures 1D,E).

## Results

Results are summarised in Table 1 and Figure 1. Total displacement of the tongue marker decreased progressively across the four admission days, reflecting reduced hyperkinetic activity. Movement variability, assessed through Poincaré analysis (SD1/SD2), showed a marked decline, indicating stabilisation of movement patterns. The number of discrete dyskinetic peaks fell from 18 on Day 1 to zero on Day 4. These glossographic findings were consistent with the clinical impression of progressive improvement documented using the UDysRS and mAIMS at each daily assessment. Patient satisfaction with the clinical outcome was high.

**Table 1:** Summary of glossographic parameters across the hospital stay.

Day	Total tongue displacement length	Range	Mean	Std	Peaks	SD1	SD2	Total vector length	Range	Mean	Std
1	73,935	215.53	95.65	57.15	18	5.85	80.55	29,719	133.39	38.45	21.31
2	40,004	106.71	51.42	29.40	3	1.13	41.54	10,323	63.59	13.27	10.93
3	13,987	51.76	24.20	13.36	1	0.61	18.87	3,612	23.82	6.25	5.16
4	5,782	18.99	7.43	4.61	0	0.46	6.49	2,540	12.02	3.26	2.66

Total vector length is the sum of all vectors between the tongue and forehead, nose, and chin. Values are given in pixels. STD, standard deviation.

## Discussion

This case report illustrates the potential of glossography as a precise and objective tool for continuous orofacial dyskinesia assessment in PD. The progressive reduction in all glossographic metrics across 4 days of hospital admission aligned with clinical assessment scores and patient-reported improvement, supporting the validity of this approach as a complement to standard rating scales.

The implications of glossography extend beyond orofacial dyskinesias in PD. Tongue dystonia – a focal dystonia characterised by involuntary, sustained or repetitive tongue contractions – represents a particularly compelling application, given the current absence of objective kinematic measurement tools in clinical practice. Further potential applications include tardive dyskinesia in patients on antipsychotic medications, oro-mandibular dystonia, and quantification of orofacial involvement in Huntington’s disease and motor neurone disease. In all of these conditions, tongue movement abnormalities are clinically significant yet currently assessed only by subjective observation.

Beyond hospital-based practice, glossography’s reliance on standard video technology makes it particularly suitable for remote monitoring. In tertiary-to-secondary referral systems, neurologists at specialist centres could remotely guide medication adjustments for patients in secondary hospitals without requiring physical transfer. This is especially relevant for patients in rural or underserved areas with limited access to movement disorder specialists.

Glossography may also find application in space medicine, where astronauts on long-duration missions face neurological deconditioning and have no access to specialist neurology. A non-contact, camera-based tool for monitoring orofacial motor function could offer mission medical officers an objective means of detecting early neurological change in environments where wearable sensors and specialist equipment are impractical to deploy.

The study is not without limitations. Most importantly, this report is based on a single patient, and all statistical analyses are derived from repeated measurements within that individual. As such, the findings should be interpreted strictly as proof-of-concept, and no generalisable conclusions regarding the performance or clinical utility of glossography can be drawn at this stage. The observed correlations between glossographic parameters and clinical improvement require validation in larger, prospective cohorts across diverse patient populations before the technique can be considered for broader clinical application. Computer vision analysis is also time- and resource-intensive, which may exceed the logistical capabilities of many centres. Total displacement metrics are video-duration-dependent and may be biased by patient head repositioning. A truncated recording (as occurred on Day 3 due to a technical issue) could skew total vector length trends. Further, the evidence base for how these parameters should influence clinical decision-making, over and above the judgement of an experienced physician, remains limited. Future research should focus on multi-patient validation studies, testing glossography in ambulatory and home-based settings, and incorporating multimodal data such as speech analysis to further enhance diagnostic accuracy.

## Data Availability

The raw data supporting the conclusions of this article will be made available by the authors, without undue reservation.
